# Clinical and functional remission: even though biologics are superior to conventional DMARDs overall success rates remain low – results from RABBIT, the German biologics register

**DOI:** 10.1186/ar1933

**Published:** 2006-04-05

**Authors:** Joachim Listing, Anja Strangfeld, Rolf Rau, Jörn Kekow, Erika Gromnica-Ihle, Thilo Klopsch, Winfried Demary, Gerd-Rüdiger Burmester, Angela Zink

**Affiliations:** 1 German Rheumatism Research Centre, Berlin, Germany; 2 Evangelisches Fachkrankenhaus, Ratingen, Germany; 3 University of Magdeburg, Magdeburg, Germany; 4 Rheumaklinik Berlin-Buch, Berlin, Germany; 5 Wilhelm-Kulz-Street 15, Neubrandenburg, Germany; 6 Bahnhofsalle 3-4, Hildesheim, Germany; 7 Charité University Hospital, Berlin, Germany

## Abstract

We investigated the frequency of remission according to the disease activity score (DAS28) definition, modified American Rheumatology Association (ARA) criteria, and the frequency of an achievement of a functional status above defined thresholds ('functional remission', 'physical independence') in rheumatoid arthritis (RA) patients treated with either biologics or conventional DMARDs. We used the data of a prospective cohort study, the German biologics register RABBIT (German acronym for Rheumatoid Arthritis – Observation of Biologic Therapy) to investigate the outcomes in RA patients with two or more DMARD failures who received new treatment with biologics (BIOL; *n* = 818) or a conventional DMARD (*n* = 265). Logistic regression analysis was applied to adjust for differences in baseline risks. Taking risk indicators such as previous DMARD failures or baseline clinical status into account, we found that biologics doubled the chance of remission compared to conventional DMARD therapies (DAS28 remission, adjusted odds ratio (OR) 1.95 (95% confidenece interval (CI) 1.2–3.2)); ARA remission, OR 2.05 (95% CI 1.2–3.5)). High remission rates (DAS28 remission, 30.6%; ARA remission, 16.9%) were observed in BIOL patients with a moderate disease activity (DAS28, 3.2 to 5.1) at the start of treatment. These rates decreased to 8.5% in patients with DAS28 > 6. Sustained remission at 6 and 12 months was achieved in <10% of the patients. Severely disabled patients (≤50% of full function) receiving biologic therapies were significantly more likely to achieve a status indicating physical independence (≥67% of full function) than controls (OR 3.88 (95% CI 1.7–8.8)). 'Functional remission' (≥83% of full function) was more often achieved in BIOL than in controls (OR 2.18 (95% CI 1.04–4.6)). In conclusion, our study shows that biologics increase the chance to achieve clinical remission and a status of functional remission or at least physical independence. However, temporary or even sustained remission remain ambitious aims, which are achieved in a minority of patients only.

## Introduction

Considering all available therapeutic options, today's treatment of rheumatoid arthritis (RA) differs substantially from what it was a decade ago. The main objectives in the treatment of RA are now to induce remission, to prevent joint destruction and to enable the patient to lead a full life. The efficacy of infliximab, etanercept, adalimumab, and anakinra was demonstrated in randomized controlled trials in the majority of RA patients enrolled [1-5]; however, less is known regarding the effectiveness of these drugs in daily rheumatological care. Standard patients differ from those enrolled into randomized controlled trials [6] with regard to disease severity, treatment history, and comorbidity. Randomized clinical trials usually focus on demonstrating relative rather than absolute improvement. In the treatment of individual patients, however, it is crucial to reach absolute targets like remission, low inflammatory activity, no pain, or a high functional capacity [7].

From the perspective of the patient, absence of pain and functional ability are the major goals. Functional independence, if not even normal function, are, therefore, major benefits that patients expect from novel therapies.

Taking physician-related and patient-related outcomes into account, two types of treatment outcomes are investigated: the percentage of patients who achieve remission according to either the disease activity score [8], or to modified American Rheumatology Association (ARA) criteria [9]; and the percentage of patients who reach either functional independence in daily activities after having been severely disabled (help dependent) at baseline or achieve (nearly) normal function ('functional remission') after a previous functional status of below physical independence at baseline. We used the data of a prospective cohort study, the German biologics register RABBIT (German acronym for Rheumatoid Arthritis – Observation of Biologic Therapy) for patients on biologic treatments as well as those on conventional disease modifying anti-rheumatic drug (DMARD) therapy.

## Materials and methods

### Patients

Patients aged 18 to 75 years meeting the American College of Rheumatology criteria for RA who were enrolled into RABBIT between 1 May 2001 and 31 December 2003 were eligible for the following analysis. RABBIT is an ongoing long-term prospective cohort study of RA patients treated with biologics or conventional DMARDs in daily rheumatological care. Patients are eligible as 'cases' if a new treatment with infliximab, etanercept, anakinra (since January 2003) or adalimumab (since September 2003) is started and as 'controls' if a conventional DMARD therapy is started after the failure of at least one other DMARD. Patients were required to give written informed consent at the time of enrolment. (for further details, see [10,11]). For better comparability of the groups, we excluded those patients from the following analysis who had only one DMARD failure, no failure of a methotrexate (MTX) therapy, who received the new treatment ≥1 day before study entry or who had a low disease activity at baseline (disease activity score based on 28 joint count (DAS28 [12]) <3.2).

The visit at 12 months was necessary for the assessment of the outcome. We excluded patients who missed the 12 months' visit (*n* = 179) because those patients did not differ significantly from patients who attended at 12 months (*n* = 1,083) with respect to important patient characteristics at baseline (age, number of DMARD failures, DAS28, joint counts, functional status). This applied to cases as well as to controls.

### Assessments

At baseline and at 3, 6, and 12 months' of follow-up, the treating rheumatologist recorded a 28 joint count of tender (TJC) and swollen (SJC) joints, erythrocyte sedimentation rate (ESR; Westergren method), C-reactive protein (CRP), morning stiffness, DMARD and/or biologic therapy, including details of start/end, reasons for treatment termination, concomitant therapies with glucocorticoids, non-steroidal anti-inflammatory drugs (NSAIDs), and adverse events. Patients self-assessed their pain, general health or fatigue on a numerical rating scale of 0 to 10. Disability was assessed by means of the Hannover Functional Status Questionnaire (Funktionsfragebogen Hannover, FFbH). The FFbH measures limitations in activities of daily living and it is comparable to the Health Assessment Questionnaire (HAQ). Scores are given in percent of full function (range 0 to 100) and can be transformed into HAQ values [13-15].

### Endpoints

Endpoints were remission according to DAS28 or to modified ARA criteria [9,16] as well as physical independence and 'functional remission'.

Clinical remission was defined as DAS28 <2.6 (DAS28 remission) [8] and according to the following modification of the ARA criteria [16]: 4 out of 5 criteria had to be fulfilled at one point in time: no tender joints; no swollen joints; ESR <30 mm/h for females or <20 mm/h for males; morning stiffness <15 minutes; pain ≤1 on a 0 to 10 scale.

'Physical independence' was defined as FFbH ≥67. This cut off point was used according to the study of Westhoff and colleagues [13], which showed that patients with an FFbH of ≥67 can be expected to be physically independent. 'Functional remission' was defined as FFbH >83. This cut off point was derived from data from 12,303 RA patients recorded in the German rheumatologic database [17] in 2003. We selected patients who were rated as Steinbrocker's functional class I by the rheumatologist [18] and who in addition rated their functional disability on a 0 to 10 numerical rating scale (0 = best functional status) as 0 or 1. More than 90% of these 1,041 patients had an FFbH >83.

The objective was to investigate the frequency of endpoints achieved at 12 months and the frequency of achievements sustained over a six month period. We excluded patients with a low disease activity (DAS28 <3.2) before start of treatment and we performed subgroup analyses with respect to the functional outcome. Changes in the treatment were not taken into account, as we did not aim to compare drugs directly.

### Statistics

Patients who received treatment with biologics had a more severe and more active disease than those receiving conventional DMARD therapies. Therefore, multivariate logistic regression was applied to adjust for confounding by indication. The adjustment was done at two consecutive stages.

At the first stage, a logistic regression estimate of the likelihood (propensity score) of being treated with biologics was made for each patient using baseline characteristics. This calculation was based on recent findings [10]. To estimate the propensity score, the final model included age, gender, number of previous DMARDs, DAS28, ESR, FFbH, and osteoporosis as well as previous treatment with cyclosporine A as additional markers of disease severity. The propensity score model fit well. There was no significant difference between observed and predicted frequencies by deciles at risk (Hosmer-Lemeshow test, *p *= 0.25). Furthermore, there was a complete overlap of the propensity scores of patients treated with biologics (BIOL) and patients receiving conventional DMARD treatment (control group; CON). It was possible, therefore, to use the propensity score as an additional risk factor of remission or functional outcome. This approach allows the adjustment for confounding by indication, which is for removing the bias in the outcome due to the covariates included in the propensity score [19].

At the second stage, logistic regression was applied to investigate which factors predicted remission or the two functional endpoints (independence and functional remission). The propensity score was included in the models as one prognostic factor. The final multivariate logistic regression models were identified by stepwise regression. These models were used for the calculation of adjusted odds ratios (ORs).

In a second approach we used the propensity score (scale: 0 to 1) to match cases (BIOL) and controls directly [19]. For each pair, a maximal difference of 0.05 propensity score units was allowed.

In the univariate and multivariate analyses, 98% of the patients had complete baseline data for all parameters considered. Outcome parameters were complete in 93% to 97% of the patients. Therefore, no imputation technique for missing data was applied. The Kruskal-Wallis test, Mann-Whitney test, and chi-square test were used to compare the baseline characteristics of the patients. The McNemar test was applied to compare frequencies between different time points and to compare the remission rates in the matched pairs analysis.

## Results

### Baseline characteristics

A total of 1,083 patients fulfilled the inclusion criteria. Among these, 818 patients had started treatment with biologics (BIOL) and 265 had started a new DMARD therapy at baseline (CON) (Table 1). The BIOL patients were younger (*p *< 0.0001) and had a significantly more active disease (DAS28, *p *< 0.0001; SJC, *p *< 0.0001; CRP, *p *< 0.0001). Furthermore they were more limited in activities involved in daily living (mean FFbH 53.1% of full function in the BIOL group versus 61.4% in the CON group, *p *< 0.0001; Table 1). At enrolment the treatment was changed in all patients. The last treatment before this change included MTX (BIOL, 56.4%; CON, 72.9%), leflunomide (BIOL, 45.0%; CON, 12.4%), sulphasalazine (BIOL, 14%; CON, 18%), other DMARDs (<15% each) or biologics (BIOL, 15.4%; CON, 3.5%). This previous treatment was applied as a combination of two or more DMARDs in 28% of the controls and 40% of the BIOL patients. In total, BIOL patients had a longer treatment history with DMARDs and significantly more previous DMARD failures (4.0 ± 1.4 versus 2.8 ± 0.9, *p *< 0.001; see also [10]).

### Remission after one year of treatment

According to the two different criteria, 10% to 16% of the patients achieved remission (Table 2), and 72% of the patients who fulfilled the modified ARA criteria for remission also fulfilled the DAS28 criteria (BIOL, 70.7%). Among those who were in DAS28 remission, however, only 54.9% also fulfilled the modified ARA criterion. Baseline DAS28 was strongly predictive of the achievement of remission (Table 2). High remission rates (DAS28 remission, 30.6%; ARA remission, 16.9%) were observed in BIOL patients with a moderate disease activity (DAS28, 3.2 to 5.1) at the start of treatment. These rates decreased to 8.5% (95% confidence interval (CI), 6.2% to 11.5%) in patients with DAS28 >6.0 for both types of remission. Highly significant negative associations between DAS28 at the start of treatment and remission at 12 months were also found by logistic regression (*p* < 0.0001) for both types of remission. These associations were stronger than those between single activity markers (SJC, TJC, ESR, CRP) and remission (Table 3). A lower functional capacity and a higher age were found to reduce significantly the chance of both types of clinical remission. This was also true if DAS28 at baseline and treatment group were controlled for (data not shown). Furthermore, patients with more than three DMARD failures, or patients with osteoporosis, had a lower chance of DAS28 remission. Patients with a disease duration ≤2 years had a higher chance of DAS28 remission, but the association did not reach statistical significance (*p *= 0.07), even if DAS28 at baseline and treatment group were controlled for (*p *= 0.06, data not shown) as the number of patients with recent onset RA was low (BIOL, *n* = 46; CON, *n *= 17).

The multivariate logistic regression models resulted in a doubled chance of remission (OR = 1.95 for DAS28 remission (*p *= 0.006); OR = 2.05 for ARA remission (*p *= 0.007)) for patients receiving biologics compared to those treated with conventional DMARDs (Table 3). The propensity score remained significant in both models, indicating that patients who had a high likelihood of being treated with biologics had a significantly lower *a priori *chance of remission.

Ninety-four percent of the BIOL patients were treated with anti-tumor necrosis factor (TNF) agents. Therefore, the adjusted ORs changed only slightly if the subgroup of patients receiving anti-TNF agents were compared with controls separately (Table 3). The ORs for the anakinra patients were lower, but they have to be interpreted with caution as the number of anakinra patients was small. We also compared the subgroup of patients receiving anti-TNF agents alone with the subgroup of patients treated with anti-TNF agents in combination with MTX. Taking prognostic factors of remission into account, no significant differences in the remission rates (DAS28 remission, *p *= 0.76; ARA remission, *p *= 0.87) were found.

The results found in the matched pairs analysis were very similar to those found by multivariate logistic regression with the propensity score as one covariate. There were 193 pairs who fulfilled the matching criteria. The pairs had similar patient characteristics at the start of treatment (Table 4). There was a highly significant difference in the remission rates between the treatment groups (DAS28 remission, OR 2.14 (95% CI 1.29–3.67); remission according to the modified ARA criteria, OR 2.00 (95% CI 1.08–3.72)).

### Sustained remission

In patients who achieved remission, a considerable risk of relapse some months later was observed. Only 44/102 (43.1%: BIOL, 32/72 (44.4%); CON, 12/30 (40%)) who were in remission after six months according to the ARA criteria remained in remission at 12 months. The corresponding figures for DAS28 remission are shown in Table 5. In total, sustained remission rates of 7.7% for DAS28 and 4.5% for ARA remission were found in patients receiving biologics.

As shown in Table 5, 24 cases were not in remission but had a DAS28 <3.2 at 12 months, while 35 patients (26.3%) deteriorated to a state of moderate or high disease activity. In 5 (BIOL, *n* = 3) of these 35 patients, the treatment was stopped between months 6 and 12 because of adverse events or non-compliance; therefore, treatment termination did not explain the increase. By logistic regression the number of previous DMARD failures was identified as a significant (*p* = 0.02) risk factor for change from remission to moderate or high disease activity. Patients with 4 to 5 (>5) DMARD failures had an OR for switching of 2.7 (CI 1.2–6.3) compared to patients with two or three failures; 4.0 (CI 1.1–15.0) for patients with >5 DMARD failures. This risk factor also explained the difference in the crude rates between BIOL and CON patients (Table 5). Adjusted for this factor, no difference (*p *= 0.99) between the groups was found.

Some patients also switched from no remission to remission, resulting in an increase in the DAS28 remission rates between months 6 and 12 (BIOL, 13.5% to 16.3% (*p *= 0.07); CON, 12.5% to 15.3% (*p *= 0.47)); however, this increase was not significant.

### Functional outcome

The number of functionally independent patients (FFbH ≥67) increased from 270 (34.2%) at start of treatment to 394 (49.9%) at 12 months (*p* < 0.001) in the BIOL group and from 122 (47.7%) to 155 (60.5%) (*p* < 0.001) in the control group. In the subgroup of patients with severe disability at baseline (FFbH ≤50 or HAQ >1.75) 78/363 (21.5%) of the BIOL compared to only 8/85 (9.4%) of the CON patients (*p* = 0.01) achieved at least functional independence. This difference between BIOL and CON patients was even higher when other risk factors of functional outcome were taken into account. Adjusted for baseline functional capacity, comorbid conditions and number of DMARD failures, an approximately four times higher chance (BIOL versus CON, OR = 3.88; anti-TNF group versus CON, OR = 4.08) of achieving functional independence was found (Table 6).

At the start of treatment, 61 (19.9%) of the controls but only 111 (11.6%) of the BIOL patients had a functional status of >83% of full function ('functional remission'; *p* < 0.001). These percentages increased to 30.1% in CON and 26.7% in BIOL patients at 12 months. In patients with a functional status below physical independence (<67) at baseline, functional remission was achieved in 12 (8.3%) of the controls but in 73 (13.2%) of the BIOL patients. In patients with a FFbH <67, however, BIOL patients differed significantly with respect to important predictors of functional remission from controls at baseline. They had a significantly worse (*p* = 0.01) functional capacity, significantly more DMARD failures, a significantly higher disease activity (DAS28, SJC, TJC, pain), and significantly higher propensity scores. After adjustment for baseline status using multivariate logistic regression, a significant difference between BIOL and CON was found (Table 6). In addition to the functional status at baseline and the treatment, the propensity score, age, and pain remained significant predictors of functional remission (Table 6). A better functional outcome was in particular found in patients receiving etanercept, infliximab or adalimumab, whereas the outcome in anakinra patients was similar to controls (Table 6).

The chance to maintain functional remission or physical independence was higher than the chance to maintain clinical remission. Most of the severely disabled patients (85.4%) who achieved an FFbH ≥67 at 6 months maintained this functional status at 12 months. Likewise, 77.2% of the patients who were in functional remission at 6 months achieved this status also at 12 months.

## Discussion

The introduction of biologic agents has substantially increased the options for treatment of RA and significantly improved the outcome. To achieve a status of low disease activity or even remission has become a realistic goal of treatment. Interestingly, this is the case not only for patients treated with these new agents but also for patients treated with conventional DMARDs. This study was conducted to investigate the remission rates according to two different criteria and the achievement of two endpoints for functional outcome (functional remission and functional independence) in a cohort of patients treated with biologics in daily practice and to compare these results with a control group of patients who changed their conventional DMARD treatment because of inefficacy or intolerance.

Of the BIOL patients, 16% achieved a DAS28 <2.6 at 12 months and 13% achieved remission according to the modified ARA criteria. The figures in the control group were 15% and 10%, respectively. Adjusting for differences in the case mix, we found that biologics doubled the chance of remission in active RA patients treated in routine care.

Sustained remission over a longer period of time is difficult to achieve [8,20,21]. Approximately half of our patients in remission at six months relapsed at 12 months, although they were under continuing rheumatologic care. This proportion is high, but it is in agreement with the findings of others [8,20,21]. For this reason, the original ARA criterion is likely to be stronger than the DAS28 criterion, since it requires ongoing remission for at least two months and also a complete absence of symptoms in the feet, which are not included in the calculation of the DAS28. These differences are more important [22] than the calculation of other cutoff values for the DAS28 remission as proposed by others [16,23,24].

To evaluate differences in remission rates, the risk profile of the patients treated had to be taken into account. Disease activity at start of treatment [21,25], disease severity, age, previous DMARD failures and comorbidities [26] indicating disease severity (for example, corticoid induced osteoporosis) were found to influence the achievement of remission. The DAS28 at baseline was the strongest predictor of remission in our data, more important than single joint counts or ESR. The chance of remission decreased with an increasing DAS28. We found that in addition to the DAS28, markers of disease severity such as rheumatoid factor [21,25], disability [21] or osteoporosis were significantly associated with the outcome. Higher rates of remission were found in patients with up to two years of disease duration. As the number of patients with early RA was too small in our data, this difference did not reach statistical significance. However, the small number of patients with early disease likely explains the differences between the remission rates found in this study and the distinctly higher rates found by others in recent onset RA [27,28]. Our remission rates at 12 months are comparable to those found in routine care (16% in [29] and 14% in [30]) or under treatment with etanercept alone or MTX alone (18% and 17%, respectively) [20]. Van der Heijde and colleagues [20] reported higher DAS28 remission rates (38%) in patients receiving a combination therapy of etanercept and MTX. Of note, patients enrolled into the TEMPO trial (Trial of Etanercept and Methotrexate with Radiographic Patient Outcomes) [20,31] had a higher *a priori *chance of remission because of a lower number of previous DMARD failures (2.3 on average; no failure of a MTX therapy) and a lower percentage of positive rheumatoid factor (76%) than our patients [31]. We found higher treatment continuation rates in patients treated with anti-TNF agents in combination with MTX compared to those receiving anti-TNF drugs alone [10], but we did not find any significant differences in the remission rates between both subgroups. More detailed analyses may be needed to determine the reasons for this finding; these analyses were beyond the scope of this paper.

Grigor and colleagues [29] showed that intensive care with a tight control of disease activity can lead to very high DAS remission rates (65%). The fact that in comparison to our patients, their patients had better preconditions regarding predictors of remission, such as disease duration or previous DMARD combination therapy, still does not provide a feasible explanation for the large difference in the remission rates. Therefore, further evaluation of this treatment strategy is needed.

It has been shown in randomized controlled trials that biologics can effectively improve function [2-5,32,33]; however, most of these trials excluded severely disabled patients [1,2,5,31,32]. This is in contrast to daily rheumatologic care in which severely disabled patients are preferred candidates for biologic treatment. Our findings suggest that especially severely disabled patients benefit from treatment with biologics. In these patients, the chance to achieve physical independence was found to be approximately four times higher if treated with biologics compared to conventional DMARDs. Furthermore, sub-sample patients who were below the threshold of physical independence at baseline were significantly more likely to achieve functional remission when a treatment with biologics was chosen.

## Conclusion

Our results complement the knowledge from clinical trials. We found that biologics, in particular anti-TNF agents, increase the chance to achieve clinical remission and a status of functional remission or at least physical independence in RA patients treated in routine care. Furthermore, in severely disabled patients with long-standing disease, significant improvement with the ability to live an independent life can be achieved by a treatment with etanercept, infliximab or adalimumab. However, temporary or even sustained remission remain ambitious aims, which are achieved in a minority of patients only.

## Abbreviations

ARA = American Rheumatology Association; BIOL = patients treated with biologics; CI = confidence interval; CON = patients receiving conventional DMARD treatment (control group); CRP = C-reactive protein; DAS28 = disease activity score based on 28 joint counts; DMARD = disease modifying anti-rheumatic drugs; HAQ = Health Assessment Questionnaire; ESR = erythrocyte sedimentation rate; FFbH = Funktionsfragebogen Hannover (Hannover Functional Status Questionnaire); MTX = methotrexate; OR = odds ratio; RA = rheumatoid arthritis; RABBIT = German biologics register (German acronym for Rheumatoid Arthritis – Observation of Biologic Therapy); SJC = 28-joint count of swollen joints; TJC = 28-joint count of tender joints; TNF = tumor necrosis factor.

## Competing interests

The authors declare that they have no competing interests.

## Authors' contributions

JL: data analysis, statistical evaluation, writing of the manuscript, study coordination, substantial contribution to conception and design. AS: responsible study physician, study coordination, writing of the manuscript. RR and JK: contribution to conception and design, involved in drafting the manuscript, acquisition and interpretation of data. EG, TK, WD, and GRB: investigators who made substantial contributions to acquisition of data. AZ: idea, principal investigator, substantial contribution to conception and design, writing of the manuscript. All authors read and approved the final manuscript. AL and AS contributed equally to this work.

## Figures and Tables

**Table 1:** Baseline characteristics.

Characteristic	Etanercept	Infliximab	Adalimumab	Anakinra	Biologics total	Control
N	397	255	121	45	818	265
Age	53.2 (12.5)	54.0 (12.3)	53.8 (12.4)	56.2 (11.2)	53.7 (12.3)	57.4 (10.3)
Female (%)	79.1	71.0	85.1	73.3	76.6	83.8
Disease duration (years), median (IQR)	10 (6–18)	9 (5–14)	12 (7–19)	13 (7–21)	10 (6–17)	9 (5–17)
Rheumatoid factor positive (%)	82.1	83.5	84.3	77.8	82.2	77.7
Swollen joint count	10.2 (6.2)	11.3 (6.4)	9.6. (6.4)	10.9 (6.6)	10.5 (6.3)	8.2 (5.3)
Tender joint count	13.0 (7.3)	13.0 (7.4)	12.3 (7.4)	13.6 (7.3)	12.9 (7.3)	10.5 (6.9)
ESR (mm/h), median (IQR)	33 (20–54)	32 (18–54)	30 (17–44)	36 (22–51)	32 (18–53)	23 (14–37)
CRP (mg/l), median (IQR)	20 (7–42)	18 (7–48)	18 (7–32)	21 (6–36)	18 (7–42)	12 (4–27)
DAS28	6.1 (1.1)	6.2 (1.2)	5.9 (1.2)	6.3 (1.0)	6.1 (1.1)	5.5 (1.1)
FFbH	53.4 (22.9)	52.8 (20.9)	53.2 (25.1)	52.7 (23.3)	53.1 (22.8)	61.4 (22.4)
Number of previous DMARDs	3.9 (1.3)	3.9 (1.4)	4.4 (1.3)	4.3 (1.6)	4.0 (1.4)	2.8 (0.9)
Current treatment DMARD (%)						
MTX alone	33.0	67.5	43.0	60.0	46.7	1.5
Other single DMARD	11.9	14.5	8.3	8.8	12.0	29.8
Combination of two DMARDs	5.3	8.6	5.8	4.4	6.4	57.3
Combinations of three DMARDs	1.3	1.6	1.6	0.0	1.3	11.3
No DMARD	48.6	7.8	41.3	26.7	33.6	0
Biologic dose/week (mg)^a^	49.7 (4.1)	4.0 (1.9)	21.5 (7.0)	700 (0)	-	-
Comorbidity (%)	72.6	68.5	64.5	71.1	70.0	72.8
Osteoporosis (%)	30.2	27.9	22.3	35.6	28.6	18.5

Values are means and standard deviations if not otherwise specified. CRP, C-reactive protein; DAS28, disease activity score based on 28 joint counts; DMARD, disease modifying anti-rheumatic drugs; ESR, erythrocyte sedimentation rate; FFbH, Funktionsfragebogen Hannover (Hannover Functional Status Questionnaire); IQR, inter quartile range. ^a^Etanercept, adalimumab and anakinra, mean dosage/week; infliximab, mean dosage/8 weeks and kg body weight, time intervals between the applications were taken into account.

**Table 2:** Patients in remission at 12 months according to DAS28 and to modified ARA criteria.

Baseline status	Patients in DAS28 remission, *n* (%)		Patients in modified ARA remission, *n* (%)	
DAS28	BIOL (*n* = 775)	CON (*n* = 255)	BIOL (*n* = 764)	CON (*n* = 256)

>3.2 to 4.0	16 (45.7)	8 (33.3)	9 (26.5)	4 (16.0)
>4.0 to 5.1	33 (26.4)	16 (23.2)	18 (14.3)	10 (14.5)
>5.1 to 6.0	40 (22.1)	10 (13.5)	38 (20.9)	8 (10.8)
>6.0 to 7.0	28 (11.1)	4 (6.3)	25 (10.1)	3 (4.8)
> 7.0	9 (5.0)	1 (4.0)	11 (6.3)	1 (3.8)
Total	126 (16.3)	39 (15.3)	101 (13.2)	26 (10.2)

ARA, American Rheumatology Association; BIOL, patients treated with biologics; CON, patients receiving conventional DMARD treatment (control group); DAS28, disease activity score based on 28 joint counts.

**Table 3:** Odds ratios (OR) of remission at 12 months.

	Remission according to DAS28 (DAS28 <2.6)				Remission according to modified ARA criteria			
	Univariate		Multivariate		Univariate		Multivariate	

	OR	95% CI	Adj. OR	95% CI	OR	95% CI	Adj. OR	95% CI

DAS28	0.53	0.45–0.62	0.46	0.35–0.61	0.69	0.59–0.82		
Propensity score (scale: 0 to 1)^a^	0.86	0.80–0.93	0.89	0.80–0.99	0.92	0.85–1.00	0.84	0.75–0.94
Rheumatoid factor positive	0.51	0.35–0.75			0.68	0.44–1.06		
ESR^b^	0.75	0.68–0.82			0.92	0.85–1.00		
CRP^b^	0.94	0.89–1.00			0.96	0.90–1.01		
Swollen joint count	0.93	0.91–0.96			0.96	0.93–0.99		
Tender joint count	0.94	0.92–0.97	1.05	1.01–1.09	0.94	0.92–0.97		
FFbH^b^	1.26	1.17–1.37			1.21	1.11–1.32	1.13	1.03–1.25
No. of previous DMARD								
4 to 5 versus 2 to 3	0.68	0.47–0.97			0.74	0.50–1.10		
≥6 versus 2 to 3	0.47	0.24–0.91			0.53	0.26–1.10		
Female	0.88	0.59–1.31			1.06	0.67–1.69		
Age^b^	0.68	0.59–0.78	0.74	0.62–0.87	0.72	0.62–0.84	0.72	0.61–0.85
Disease duration (years)	0.98	0.96–1.00			0.99	0.96–1.00		
Disease duration ≤2 years versus >2 years	1.86	0.97–3.58			0.81	0.32–2.09		
Any comorbidity yes versus no	0.52	0.37–0.73			0.68	0.46–1.01		
Osteoporosis yes versus no	0.38	0.23–0.61	0.51	0.30–0.87	0.69	0.44–1.10		
BIOL versus CON	1.08	0.73–1.59	1.95	1.20–3.19	1.35	0.85–2.13	2.05	1.19–3.52
Anti-TNF agents versus CON	1.10	0.74–1.63	1.97	1.20–3.21	1.39	0.88–2.20	2.09	1.22–3.59
Anakinra versus CON	0.71	0.26–1.91	1.66	0.56–4.91	0.68	0.20–2.36	1.19	0.33–4.34

Results of different univariate and multivariate logistic regression analyses. Final multivariate models were found by stepwise regression. ^a^Odds ratios (ORs) refer to 0.1 units of the propensity score. ^b^The ORs refer to 10 units of the parameter. Adj. OR, adjusted odds ratio; ARA, American Rheumatology Association; BIOL, patients treated with biologics; CI, confidence interval; CON, patients receiving conventional DMARD treatment (control group); CRP, C-reactive protein; DAS28, disease activity score based on 28 joint counts; DMARD, disease modifying anti-rheumatic drugs; ESR, erythrocyte sedimentation rate; FFbH, Funktionsfragebogen Hannover (Hannover Functional Status Questionnaire); TNF, tumor necrosis factor.

**Table 4:** Matched pairs analysis: patients' characteristics and rates of remission at 12 months.

	BIOL	CON	*p* value^a^
N	193	193	
Characteristics at baseline			
Age	54.7 (12.1)	55.9 (10.3)	0.50
Female (%)	83.4	79.8	0.43
Rheumatoid factor positive (%)	82.4	79.8	0.60
Swollen joint count	8.7 (6.1)	8.8 (5.5)	0.59
Tender joint count	11.3 (7.3)	11.4 (6.8)	0.61
ESR (mm/h), median (IQR)	22 (13–40)	24 (15–40)	0.37
CRP (mg/L), median (IQR)	14 (6–28)	12 (5–29)	0.19
DAS28	6.1 (1.1)	6.1 (1.1)	0.54
FFbH	59.3 (21.1)	58.9 (22.6)	0.92
No. previous DMARDs	3.1 (1.1)	3.0 (1.0)	0.74
Propensity score	0.63 (0.19)	0.63 (0.19)	-
Remission at 12 months, *n *(%)			
DAS28 Remission	48 (24.9%)	24 (12.4%)	0.004
Remission according to modified ARA criteria	31 (16.1%)	16 (8.3%)	0.036

In this analysis pairs of BIOL and CON patients who differed by less than 0.05 on the propensity score scale were included. Values are means and standard deviations if not otherwise specified. ^a^Mann-Whitney test and chi-square test were applied as appropriate for the comparision of the baseline characteristics and McNemar test for the comparison of the remission rates. ARA, American Rheumatology Association; BIOL, patients treated with biologics; CON, patients receiving conventional DMARD treatment (control group); CRP, C-reactive protein; DAS28, disease activity score based on 28 joint counts; DMARD, disease modifying anti-rheumatic drugs; ESR, erythrocyte sedimentation rate; FFbH, Funktionsfragebogen Hannover (Hannover Functional Status Questionnaire); IQR, inter quartile range.

**Table 5:** Disease activity at 12 months for patients in DAS28 remission at 6 months.

Disease activity at 12 months	BIOL, *n* (%)	CON, *n* (%)
In remission (DAS28 <2.6)	56 (54.9%)	18 (58.1%)
Low disease activity (DAS28 <3.2)	17 (16.7%)	7 (22.6%)
Moderate or high disease activity (DAS28 >3.2)	29 (28.4%)	6 (19.4%)
Total	102	31

BIOL, patients treated with biologics; CON, patients receiving conventional DMARD treatment (control group); DAS28, disease activity score based on 28 joint counts.

**Table 6:** Adjusted odds ratios of physical independence and normal functionat 12 months.

	Achievement of FFbH ≥67 (physical independence)				Achievement of FFbH ≥83 (functional remission)			
	Univariate		Multivariate		Univariate		Multivariate	

	OR	95% CI	Adj. OR	95% CI	OR	95% CI	Adj. OR	95% CI

FFbH^a^	1.54	1.23–1.92	1.51	1.20–1.90	1.84	1.51–2.23	1.85	1.50–2.29
Propensity score (scale: 0 to 1)^b^	0.96	0.85–1.09			0.93	0.84–1.04	0.85	0.74–0.98
Rheumatoid factor positive	0.99	0.51–1.90			0.67	0.38–1.16		
Number of previous DMARDs								
4 to 5 versus 2 to 3	0.73	0.44–1.20	0.67	0.39–1.15	0.52	0.31–0.86		
≥6 versus 2 to 3	0.40	0.17–0.94	0.39	0.16–0.94	0.50	0.23–1.10		
Swollen joint count	1.03	0.99–1.07			1.01	0.97–1.05		
Tender joint count	1.01	0.97–1.04			0.98	0.95–1.01		
ESR^a^	0.96	0.86–1.06			0.95	0.86–1.05		
DAS28	1.08	0.86–1.34			0.90	0.74–1.10		
Pain (0 to 10)	1.05	0.92–1.19			1.02	0.91–1.15	1.22	1.05–1.40
Female	0.79	0.44–1.40			0.94	0.53–1.68		
Age^a^	0.70	0.56–0.88			0.62	0.51–0.76	0.64	0.51–0.80
Disease duration (years)	0.95	0.92–0.98			0.94	0.91–0.97		
Disease duration ≤2 years versus >2 years	1.21	0.39–3.78			1.59	0.64–3.96		
Any comorbidity, yes versus no	0.38	0.23–0.62	0.42	0.25–0.71	0.46	0.28–0.73		
Osteoporosis, yes versus no	0.47	0.27–0.83			0.43	0.24–0.78		
BIOL versus CON	2.63	1.22–5.69	3.88	1.71–8.79	1.68	0.89–3.19	2.18	1.04–4.57
Anti-TNF agents versus CON	2.77	1.28–6.00	4.09	1.80–9.29	1.73	0.91–3.29	2.21	1.06–4.63
Anakinra versus CON	0.57	0.07–4.83	0.79	0.09–7.20	0.82	0.17–3.88	1.33	0.26–6.94

The achievement of FFbH ≥67 was investigated only in severely disabled patients with high risk of needing external help (FFbH ≤50, cutoff point used to identify severely disabled patients with a high risk of needing external help) [13] before the start of treatment (BIOL, *n *= 452 (anti-TNF agents, *n *= 425; anakinra, *n *= 27); CON, *n *= 100); the achievement of FFbH >83 was investigated only in patients who were at any risk of needing external help (FFbH <67, cut-off point used to identify patients who were under any increased risk of needing external help) [13], (BIOL, *n *= 682 (anti-TNF agents, *n *= 643; anakinra, *n *= 39); CON, *n *= 173). Results of different univariate and multivariate logistic regression analyses. Final multivariate models were found by stepwise regression. ^a^The ORs refer to 10 units of the parameter. ^b^OR refer to 0.1 units of the propensity score. Adj. OR, adjusted odds ratio; BIOL, patients treated with biologics; CI, confidence interval; CON, patients receiving conventional DMARD treatment (control group); CRP, C-reactive protein; DAS28, disease activity score based on 28 joint counts; DMARD, disease modifying anti-rheumatic drugs; ESR, erythrocyte sedimentation rate; FFbH, Funktionsfragebogen Hannover (Hannover Functional Status Questionnaire); TNF, tumor necrosis factor.
